# Editorial: The role of astrocyte signalling pathways in ageing-induced neurodegenerative pathologies

**DOI:** 10.3389/fphys.2022.1002428

**Published:** 2022-09-02

**Authors:** Nady Braidy, Bilal Cig, Lin-Hua Jiang, Xinhua Shu

**Affiliations:** ^1^ Centre for Healthy Brain Ageing, School of Psychiatry, University of New South Wales, Sydney, NSW, Australia; ^2^ Wolfson Centre for Age-Related Diseases, School of Neuroscience, Institute of Psychiatry, Psychology and Neuroscience, King’s College London, London, United Kingdom; ^3^ Department of Physiology, School of Medicine, Kirsehir Ahi Evran University, Kirsehir, Türkiye; ^4^ Sino-UK Joint Laboratory of Brain Function and Injury of Henan Province, Department of Physiology and Pathophysiology, Xinxiang Medical University, Xinxiang, China; ^5^ A4245-Transplantation, Immunology and Inflammation, Faculty of Medicine, University of Tours, Tours, France; ^6^ Faculty of Biological Sciences, School of Biomedical Sciences, University of Leeds, Leeds, United Kingdom; ^7^ Department of Biological and Biomedical Sciences, Glasgow Caledonian University, Glasgow, United Kingdom; ^8^ School of Basic Medical Sciences, Shaoyang University, Shaoyang, China

**Keywords:** Astrocytes, neurodegenerative diseases, calcium, purines, glia

Astrocytes are a group of cells with specific morphological and functional characteristics that vary in different regions of the brain. During postnatal development, astrocytes migrate to the regions of interest in the brain. Astrocytes interact with neurons to protect, support and maintain normal cellular homeostasis during physiological ageing of the central nervous system (CNS), including the brain, spinal cord and retina. Mature astrocytes retain most of the genes expressed in progenitor cells to keep their proliferative potential. In the last few decades, astrocytes have been reported to serve an important role in age-related neurodegenerative diseases.

Ageing is complex and multifactorial and leads to alterations in several processes modulated by astrocytes including but not limited to synaptic plasticity, gap junctional communication, oxidative stress and neuroinflammation, neurotransmission, release of neurotrophic factors, and water/ion metabolic balance. Ion channels, a group of specialized membrane proteins acting as signal sensing, integrating or transducing molecules, play a vital part in regulating of numerous astrocyte signalling pathways. In the CNS, oxidative stress and neuroinflammation are two of the main pathological hallmarks associated with aging. Increased age-related oxidative stress and inflammation can contribute to development and progression of multiple neurodegenerative diseases including Parkinson’s disease (PD), Huntington’s disease (HD), Alzheimer’s disease (AD) and mild cognitive impairment (MCI). Astrocytes are also key mediators of responses to traumatic brain injury (TBI) and spinal cord injury (SCI). Impaired astrocyte function due to cellular senescence has major implications in neurodegenerative diseases and the ageing brain. The series of articles in this Research Topic review current thinking with regards to the role of astrocytes in ageing and neurodegenerative disorders, and highlight the possibility of targeting astrocytes as a therapeutic strategy for the treatment of brain disorders, and their effects on other brain cells.

The first article by Mira et al. provides a potential hypothesis of the role of astrocytes in response to TBI. Astrocytes are crucial for maintenance of the brain’s ion and water homeostasis, energy metabolism, blood brain barrier integrity and immunogenicity. TBI induces apoptosis of neurons, demyelination of oligodendrocytes and reduced axonal transport, and there are interactions between astrocytes and microglial cells and neurons which contribute to the pathobiology of TBI.

The second article by Bancroft and Srinivasan reviews the aberrant roles of astrocytic calcium signalling in PD. It is well established that astrocytes produce robust intracellular Ca2+ signals that are key regulators of astrocyte function. The identification of active intracellular Ca2+ signalling in astrocytes provided renewed insight on the role of astrocytes in neuronal function. Astrocytic Ca2+ signalling has been implicated in important physiological and pathophysiological events including the loss of dopaminergic neurons in PD and other neurodegenerative diseases.

Astrocytes are major glial cells that are involved in the maintenance of structural and functional integrity and regulate neuronal function in the CNS. Astrocytes are also involved in the regulation of neurogenesis and synaptogenesis, and maintenance of the blood brain barrier. However, controversy remains on whether astrocytes play a direct role in mediating cell death and reducing function in patients with neurodegenerative diseases. The review article by Bouvier et al. provides a translational overview on the role of astrocytes in mediating neurotoxicity in both human cell culture and murine animal models for neurodegenerative diseases. It also highlights the role of astrocytes in physiological aging and their response to pathogenic stimuli and its relationship to various disease states. These findings highlight the importance of pharmacological strategies that ameliorate or reverse astrocyte toxicity and their impact on neurodegeneration and cognitive decline in the clinic.

There is considerable evidence of the importance of astrocyte ion channels in mediating CNS homeostasis. Several ion channel proteins have been identified in astrocytes including aquaporins, transient receptor channels, adenosine triphosphate sensitive potassium channels, and P2X7 receptors. These ion channels enable astrocytes to interact with neurons and regulate synaptic transmission and neuronal plasticity. They have also been associated with increased oxidative stress, neuroinflammation and abnormal aggregation of protein linked to AD, PD and HD. The review by Wang et al. discusses recent developments and future perspectives in astrocytic ion channels, which may lead to the identification and development of novel therapeutic strategies for neurodegenerative disorders.

Astrocytes play a significant role in regulating brain energy metabolism. Their anatomical position in close proximity to blood vessels and neurons allows them to serve as an essential interface for efficient uptake of glucose from the blood, which can be further utilised in various metabolic pathways. Neuro-energetic coupling which is regulated by astrocytes via glutamate uptake induces astrocytic aerobic glycolysis which is impaired in age-related neurodegenerative disorders such as AD. As well, neurotransmitter action on astrocytes has important roles on energy metabolism. The review by Beard et al. discusses the importance of astrocytes in regulating brain energy and how impairment in astrocyte-mediated metabolic pathways is associated with brain hypometabolism. Understanding astrocyte energy metabolism may not only increase our understanding on neuron-astrocyte interactions, but also identify brain hypometabolism as a new target for the development of therapeutic strategies for the treatment of age-related disorders.

Subcellular microRNA (miRNA) localization plays crucial roles in mediating cellular development, differentiation, and migration. The review by Chu and Williams discusses the emerging role of astrocytic miRNA in regulating gene expression. Impairments in astrocytic miRNA can have a profound impact on apoptotic processes, cytokine release, immunogenicity, and cellular function, and may exacerbate the development of neuropathology and phenotype associated with neurodegenerative diseases. The review also discusses potential regulatory mechanisms at the miRNA level with potential significance for astrocyte function during brain ageing and disease.

Astrocytes and microglia exhibit complementary roles in the CNS. Apart from supporting synaptogenesis and neuronal function, astrocytes and microglia also have a supportive role on myelination, blood brain barrier regulation and angiogenesis. In response to inflammation and other pathogenic insult, microglia can mediated context-specific signals in murine models of neurodegenerative disorders, and AD in particular. The review article by Castillo et al. discusses important molecular mechanisms that regulate astrocyte-microglial communications, including signalling through cytokine release and environmental signals such as purines. Purinergic receptors including P2X4 receptor are associated with microglial activation and their expression is increased in most neurodegenerative disease states. P2X4 receptor may represent an important link between ageing and neuroinflammation.

The sum of the articles adds to our recent work in the areas of astrocyte form and function in ageing and neurodegenerative diseases. The articles in this Research Topic provide a summary of the multiple roles of astrocytes and other glial cells including microglia in the pathogenesis of neurodegenerative disorders and provide emerging evidence for astrocytes and microglial as a target for the development of therapeutic agents to attenuate neuropathological states and improve cognitive and/or locomotor function.

